# COVID-19 Prevention Behaviors among Health Staff: Data from a Large Survey in the West of Iran

**DOI:** 10.34172/jrhs.2021.43

**Published:** 2021-02-14

**Authors:** Saeid Bashirian, Salman Khazaei, Majid Barati, Ensiyeh Jenabi, Ali Reza Soltanian, Samane Shirahmadi, Akram Karimi-Shahanjarini, Sepideh Zareian, Forouzan Rezapur-Shahkolai, Babak Moeini

**Affiliations:** ^1^Social Determinants of Health Research Center, Hamadan University of Medical Sciences, Hamadan, Iran; ^2^Department of Public Health, School of Public Health, Hamadan University of Medical Sciences, Hamadan, Iran; ^3^Research Center for Health Sciences, Hamadan University of Medical Sciences, Hamadan, Iran; ^4^Autism Spectrum Disorders Research Center, Hamadan University of Medical Sciences, Hamadan, Iran; ^5^Modeling of Noncommunicable Diseases Research Center, School of Public Health, Hamadan University of Medical Sciences, Hamadan, Iran; ^6^Department of Community Oral Health, School of Dentistry, Hamadan University of Medical Sciences, Dental Research Center, Hamadan, Iran; ^7^Head of Statistics and Information Technology Management Infrastructure Department, Hamadan University of Medical Sciences, Hamadan, Iran

**Keywords:** Coronavirus, Behavior, Fear, Iran

## Abstract

**Background:** Hospital staffs are at high risk of Novel Coronavirus (2019-nCoV preventive behaviors play a peculiar role in the reduction of the incidence and mortality of this infection. Therefore, the present study aimed to assess the prevention behaviors of COVID-19 among health staff based on the Extended Parallel Model (EPPM) in western Iran.

**Study design:** It was a cross-sectional study.

**Methods:** The present study was performed in the west of Iran in April 2020. In total, 1,664 cases were enrolled in this study via multi-stage sampling. The data were collected using a questionnaire, including the demographic characteristics of participants and EPPM constructs. All analyses were conducted in Stata software (version 14) at a 5% significant level.

**Results:** As evidenced by the obtained results, 1,523 (91.53%), 1,226 (73.68%), 1,526 (91.71%), 893 (53.67%), and 862 (51.86%) of health staff wear gloves, use masks, avoid contact with others, maintain a good distance from other people, and wash their hands frequently with water and soap, respectively. In terms of using gloves and avoiding contacts with others, participants with high perceived threat had higher odds of observing health behaviors (OR= 3.14, 95% CI: 2.08, 4.73; *P*<0.001) and (OR= 3.1, 95% CI: 2.04, 4.69; *P*<0.001), respectively. In all categories of EPPM, the participants with high efficacy had higher odds of exhibiting health behaviors, compared to those with low efficacy (*P*<0.001).

**Conclusion:** The results of the present study demonstrated that health workers are expected to be at the highest level of threat and efficiency. Moreover, the findings emphasized the effectiveness of the recommended strategies in the prevention of COVID-19 disease.

## Introduction

Coronaviruses are positive-sense single-stranded RNA viruses which are assigned to four major subgroups, including alpha, beta, gamma, and delta 1. Novel Coronavirus (2019-nCoV) (the cause of COVID-19) was first identified in Wuhan Hubei province (China) and spread to numerous countries across the globe 2-4. Ample evidence has suggested that COVID-19 has a zoonotic source5. Old age, male gender, and presence of comorbidities have been recognized as the risk factors for poor prognosis of the disease ^2^. Approximately 80% of COVID-19 infections are mild or asymptomatic, 15% of cases are severe infections requiring oxygen, and 5% are critical infections requiring ventilation ^6^. The fatality rate of COVID-19 is between 3% and 4% ^6^.

 The COVID-19 is widespread and can quickly be transmitted by contact, droplets, and fomites. Consequently, public health measures, such as hand hygiene and respiratory etiquette, are necessary for infection prevention6. Therefore, identification, isolation, and patient care in the early stages are of paramount importance. The first cases of this disease (43 patients and 8 deaths) in Iran were reported between February 19 and 23, 2020 7. Health staff are on the front lines of care and treatment of COVID-19 patients and have an increased risk of exposure to this virus. Moreover, the inadequate human resources involved in the care and the high risk of health staff highlight the need for safety considerations to protect medical staff and prevent the spread of infection.

The use of N95 masks, goggles, and protective gowns as health behaviors can play a critical role in COVID-19 prevention among health staff ^8^. The Extended Parallel Process Model (EPPM) is useful for understanding adaptive behaviors in the face of unknown risks ^9^. The EPPM has been widely adopted as a framework for the prediction in a range of health-related behaviors ^10^. It also evaluates fear prediction and encourages people to perform protective behaviors ^11^. In light of the aforementioned issues, the present study aimed to assess the prediction of prevention behaviors of COVID-19 among the staff of health Centers and hospitals based on the Extended Parallel Model (EPPM) in western Iran.

## Methods

 This cross-sectional study was performed in Hamadan Province, west of Iran, in April 2020. The study population included 22% of total health staff (1,664 out of 7,500 cases). The required sample size was estimated at 1,725 cases assuming that 90% of staff follow health behaviors, considering 95% confidence interval, precision equal to 0.02, and the design effect equal to 2. Finally, 1,668 subjects were included in the study after the removal of distorted questionnaires.

 The participants were selected via multi-stage sampling (sequence of Stratified- simple random sampling) with a proportional to size weights. Firstly, 876 (52.65%) 788 (47.35%) cases were allocated to health centers and hospitals, respectively. Thereafter, we assigned them a sample size proportional to the population size of different job categories. For each job category, we received the mobile number of the employees from the relevant manager according to the allocated sample size via random sampling. The subjects received the link to the questionnaire via text messages to answer the questions. A new person was randomly replaced those who did not respond (nearly 15%). Finally, out of 1664 participants (876 health staff and 788 hospital staff) contributed to the present study.

 The protocols of the present study were approved by the Ethical Committee of Hamadan University of Medical Science (IR.UMSHA.REC.1398.1092). The inclusion criteria were as follows: 1) being a staff in the health Centers and hospitals, and b) willingness to participate in the survey. The questionnaire used in this study consisted of two sections: a) socio-demographic characteristics including age, gender, job, educational status, the source for Coronavirus information, and the use of protective measures, such as mask, goggles, and protective gowns, b) Questionnaire about predicting protective behaviors based on EPPM.

The EPPM constructs include 20 items: a) perceived susceptibility (n=2), b) perceived severity (n=3), c) self-efficacy (n=5), and d) response efficacy (n=5). In addition, there were 5 items from the health behaviors regarding the COVID-19 pandemic. The threat appraisal score is the sum of the perceived susceptibility and severity scores. Moreover, the perceived efficacy score is the sum of the response efficacy and self-efficacy ^12^.

To standardize efficacy item scores, the mean of the efficacy item scores are subtracted from each efficacy item score and then divided by the standard deviation of the efficacy scores. The same procedure is used to standardize threat scores. If standardization proves cumbersome for health care providers or practitioners, normative means and standard deviations can be calculated for a target population (e.g., college students, elderly Midwesterners, urban junior high school adolescents). This issue is addressed in the Discussion. If the obtained score is positive, the person (or audience) who completed the scale is engaging in a danger control process since the perceptions of efficacy outweigh those of threat. If the obtained score is negative, the person (or audience) who completed the scale is engaging in fear control processes since the perceptions of threat outweigh those of efficacy ^13^.

 These items of EPPM constructs are rated on a 5-point Likert scale ranging from 1=strongly disagree to 5=strongly agree. We calculated the score of each subscale by averaging the sum of its items. The preventive behaviors for COVID-19 among health staff were calculated by 5 items rated on a 3-point Likert scale (“always”, “sometimes”, and “never”, scored 2, 1, and 0, respectively). The face and content validity were performed. The validity was checked by 10 health education experts. Moreover, the reliability of the questionnaire was approved by calculating internal consistency. The Cronbach’s alpha and test-retest reliability were reported as 0.70-0.75 and 0.71- 0.82, respectively.

 Descriptive statistics were reported as number (%) and mean (SD) across demographic characteristics of the respondents. The normality assumption of the outcome variables was checked through the Shapiro-Wilk test. In terms of EPPM, four scenario-specific profiles for the EPPM were created based on the levels of the perceived threat and perceived efficacy: 1) low threat-low efficacy (LT/LE), 2) low threat-high efficacy (LT/HE), 3) high threat-low efficacy (HT/LE), and 4) high threat-high efficacy (HT/HE). The association between respondents’ demographic characteristics categories of the EPPM model was assessed using the Chi-square test. Furthermore, the mean score of protection motivation theory (PMT) constructs according to respondents’ demographic characteristics was compared using independent t-test and one-way ANOVA. Moreover, univariate logistic regression and multivariable logistic regression were performed to determine the effect of different categories of EPPM on five assessed health behaviors. Hosmer and Lemeshow strategy was used for model building and the model fitted with all variables that had a p-value less than 0.2. All statistical analyses were conducted in STATA software (version 14). A p-value less than 5% was considered statistically significant.

## Results

Out of 1664 participants (876 health centers staff and 788 hospital staff), 930 (55.89%) cases were female, and 678 (40.75%) subjects were in the age group of 30-39 years. Nearly half of them (48.02%) had work experience of more than 10 years. Regarding job status, the staff of health centers, nurses, and service personnel were more involved in the current study with 37.5%, 17.73%, and 17.01%, respectively (Table 2). The mean constructs of PMT according to gender, age group, and work history are displayed in Table 1. Females had a significantly higher score in all PMD constructs (P<0.050). The scores of perceived severity and self-efficacy were significantly different among different age groups (P=0.024); moreover, the scores of response efficacy were different according to the work history of participants (P=0.015).

**Table 1:** Mean of the constructs of protection motivation theory based on gender, age group, and work history of participants

**Variables**	**Perceived susceptibility**			**Perceived severity**			**Self-efficacy**			**Response efficacy**		
	**Mean**	**SD**	***P*** **-value**	**Mean**	**SD**	***P*** **-value**	**Mean**	**SD**	***P*** **-value**	**Mean**	**SD**	***P*** **-value**
Gender			0.001			0.001			0.023			0.001
Male	5.91	1.36		11.96	2.33		20.54	3.47		20.28	3.40	
Female	6.15	1.20		12.72	1.92		20.94	3.55		20.99	3.19	
Age group (yr)			0.201			0.024			0.024			0.882
20-29	6.05	1.17		12.58	1.87		20.47	3.77		20.6	2.23	
30-39	6.11	1.29		12.41	2.16		20.64	3.61		20.65	3.36	
40-49	6.00	1.29		12.34	2.16		21.04	3.15		20.76	3.12	
50-60	5.89	1.41		11.97	2.57		21.27	3.42		20.78	3.45	
Work history (yr)			0.547			0.216			0.162			0.015
<5	6.02	1.21		12.32	2.05		20.52	3.61		20.56	3.4	
5-10	6.11	1.43		12.57	2.14		20.90	3.64		21.04	3.11	
>10	6.04	1.24		12.37	2.20		20.86	3.41		20.59	3.32	
Total score	6.04	1.28		12.39	2.14		20.67	3.70		20.67	3.70	
Range	3 to 10			3 to 15			5 to 25			5 to 25		

Table 2 presents the associations of demographic characteristics of respondents with threat and efficacy categories regarding COVID-19. Following the EPPM, the proportion of participants with low perceived threat and efficacy (LT/LE), low threat-high efficacy (LT/HE), high threat-low efficacy (HT/LE), and high perceived threat and efficacy (HT/HE) were reported as 1.38%, 10.04%, 2.54%, and 86.06%, respectively. The proportion of high threat/high efficacy profile was higher among females (90.65% vs. 80.25% in males; P<0.001), participants with 20-29 years of age (88.61; P=0.047), and in paramedicine staff (90.09%; P=0.003).

**Table 2:** Associations of respondents' demographic characteristics with threat and efficacy categories to COVID-19 pandemic

**Variables**	**Total**	**Low threat-Low** **efficacy**		**Low threat-High** **efficacy**		**High threat-Low** **efficacy**		**High threat-High** **efficacy**		***P*** **-value**
		**Number**	**Percent**	**Number**	**Percent**	**Number**	**Percent**	**Number**	**Percent**	
Gender										0.001
Male	734	16	2.18	110	14.99	19	2.59	589	80.25	
Female	930	7	0.75	57	6.13	23	2.47	843	90.65	
Age group (yr)										0.047
20-29	395	6	1.52	27	6.84	12	3.04	350	88.61	
30-39	678	8	1.18	80	11.80	22	3.24	568	83.78	
40-49	434	4	0.92	43	9.91	5	1.15	382	88.02	
50-60	157	5	3.18	17	10.83	3	1.91	132	84.08	
Work history (yr)										0.576
<5	515	8	1.55	54	10.49	16	3.11	437	84.85	
5-10	350	3	0.86	39	11.14	11	3.14	297	84.86	
>10	799	12	1.50	74	9.26	15	1.88	698	87.36	
Job										0.003
Physicians	230	1	0.43	34	14.78	5	2.17	190	82.61	
Nurses	295	5	1.69	39	13.22	5	1.69	246	83.39	
Para-medicine	232	1	0.43	17	7.33	5	2.16	209	90.09	
Health centers	624	6	0.96	51	8.17	21	3.37	546	87.50	
Service personnel	283	10	3.53	26	9.19	6	2.12	241	85.16	

The associations between the categories of EPPM with healthy behaviors regarding the COVID-19 pandemic are presented in Table 3. In terms of using gloves and avoiding contacts with others, participants with high perceived threat had higher odds of exhibiting healthy behaviors ([OR= 3.14, 95% CI: 2.08, 4.73)], P<0.001) and ([OR= 3.1, 95% CI: 2.04, 4.69)], P<0.001), respectively. In all categories of EPPM, compared to participants with low efficacy, participants with high efficacy had higher odds of displaying healthy behaviors (P<0.001). The odds of exhibiting all five healthy behaviors were significantly higher for the high threat/high efficacy (HT/HE) and low threat-high efficacy (LT/HE) categories, compared to the low threat/low efficacy (LT/LE) category (P<0.050). Furthermore, the odds of using gloves and avoiding contact with others were significantly higher in the high threat/low efficacy (HT/LE) category, in comparison with the LT/LE category (P<0.050). In Table 4, we adjusted the associations between the categories of EPPM and healthy behaviors for gender, age, and work history. These results were similar to the crude model in Table 3.

**Figure 1:**
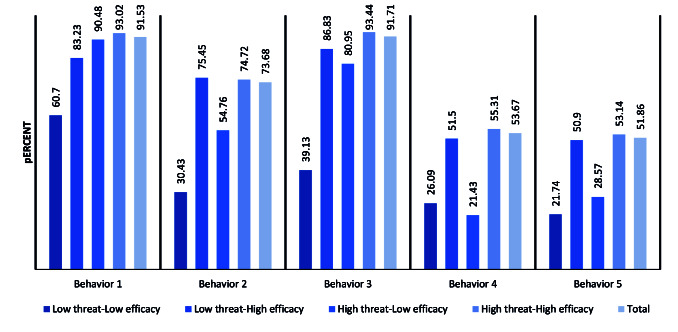
Health behaviors of health and hospital staffs regarding the prevention of COVID-19 according to the categories of the Extended Parallel Model
 (Behavior 1: use of gloves, Behavior 2: use of masks, behavior 3: avoiding contact with others, behavior 4: keep a good distance from other people (at least 1.5 meters), behavior 5: Frequent hand washing with water and soap)

The health behavior of study participants regarding the prevention of COVID-19 is illustrated in Figure 1. The results indicated that 1523 (91.53%), 1226 (73.68%), 1526 (91.71%), 893 (53.67%), and 862 (51.86%) of health staff wear gloves, use masks, avoid contact with others, maintain a good distance from other people, and wash their hands frequently with water and soap, respectively. In all five investigated behaviors, participations with LT/LE had a lower proportion of constant exhibition of these behaviors.

## Discussion

 This study aimed to determine the factors associated with preventive behaviors of COVID-19 among health staff in the west of Iran. The proportion of participations with low both perceived threat and efficacy (LT/LE), low threat-high efficacy (LT/HE), high threat-low efficacy (HT/LE), and high both perceived threat and efficacy (HT/HE) were obtained at 1.38%, 10.04%, 2.54%, and 86.06%, respectively. Moreover, health staff with low threat and efficacy had a lower rate of the constant exhibition of health behaviors.

**Table 3:** Associations between the categories of the Extended Parallel Process Model (EPPM) and health behavior regarding COVID-19 pandemic (crude model)

**EPPM categories**	**Behavior 1**		**Behavior 2**		**Behavior 3**		**Behavior 4**		**Behavior 5**	
	**OR (95% CI)**	***P*** **- v alue**	**OR (95% CI)**	***P*** **- v alue**	**OR (95% CI)**	***P*** **- v alue**	**OR (95% CI)**	***P*** **- v alue**	**OR (95% CI)**	***P*** **- v alue**
Threat										
Low	1.00		1.00		1.00		1.00		1.00	
High	3.14 (2.08, 4.73)	0.001	1.21 (0.86, 1.68)	0.260	3.1 (2.04, 4.69)	0.001	1.3 (0.96, 1.75)	0.090	1.22 (0.9, 1.65)	0.190
Efficacy										
Low	1.00		1.00		1.00		1.00		1.00	
High	2.99 (1.58, 5.65)	0.001	3.72 (2.23, 6.18)	0.001	6.86 (3.96, 11.92)	0.001	3.89 (2.16, 7.00)	0.001	3.3 (1.86, 5.87)	0.001
Combination										
Low threat-Low efficacy	1.00		1.00		1.00		1.00		1.00	
Low threat-High efficacy	3.19 (1.26, 8.09)	0.015	7.02 (2.7, 18.26)	0.001	10.25 (3.97, 26.51)	0.001	3.01 (1.13, 8.01)	0.027	3.73 (1.32, 10.52)	0.013
High threat-Low efficacy	6.11 (1.62, 23.04)	0.008	2.77 (0.94, 8.12)	0.064	6.61 (2.12, 20.62)	0.001	0.77 (0.24, 2.53)	0.670	1.44 (0.44, 4.76)	0.550
High threat-High efficacy	8.56 (3.62, 20.27)	0.001	6.76 (2.76, 16.55)	0.001	22.14 (9.34, 52.49)	0.001	3.51 (1.37, 9.44)	0.009	4.08 (1.51, 11.06)	0.006

Behavior 1: use of gloves Behavior 2: use of masks, Behavior 3: avoiding contact with others Behavior 4: keep a good distance from other people (at least 1.5 meters) Behavior 5: washing frequently hands with water and soap)

**Table 4:** Associations between the categories of the Extended Parallel Process Model (EPPM) and health behavior regarding COVID-19 pandemic (adjusted model ^a^)

**EPPM categories**	**Behavior 1**		**Behavior 2**		**Behavior 3**		**Behavior 4**		**Behavior 5**	
	**OR (95% CI)**	***P*** **-value**	**OR (95% CI)**	***P*** **-value**	**OR (95% CI)**	***P*** **-value**	**OR (95% CI)**	***P*** **-value**	**OR (95% CI)**	***P*** **-value**
Threat										
Low	1.00		1.00		1.00		1.00		1.00	
High	2.77 (1.82, 4.23)	0.001	1.24 (0.88, 1.74)	0.260	2.59 (1.68, 3.99)	0.001	1.22 (0.90, 1.66)	0.190	1.16 (0.86, 1.58)	0.330
Efficacy										
Low	1.00		1.00		1.00		1.00		1.00	
High	2.79 (1.47, 5.32)	0.002	3.66 (2.19, 6.13)	0.001	6.67 (3.77, 11.81)	0.001	3.84 (2.13, 6.92)	0.001	3.21 (1.80, 5.72)	0.001
Combine										
Low threat-Low efficacy	1.00		1.00		1.00		1.00		1.00	
Low threat-High efficacy	3.32 (1.29, 8.57)	0.013	7.40 (2.81, 19.48)	0.001	12.83 (4.74, 34.75)	0.001	2.94 (1.10, 7.83)	0.031	3.71 (1.31, 10.50)	0.013
High threat-Low efficacy	6.21 (1.62, 23.85)	0.008	3.20 (1.08, 9.56)	0.036	7.69 (2.34, 25.23)	0.001	0.71 (0.22, 2.34)	0.580	1.42 (0.43, 4.71)	0.570
High threat-High efficacy	7.86 (3.27, 18.92)	0.001	7.30 (2.94, 18.14)	0.001	22.52 (9.09, 55.80)	0.001	3.25 (1.27, 8.31)	0.014	3.88 (1.43, 10.54)	0.008

(Behavior 1: use of gloves, Behavior 2: use of masks, behavior 3: avoiding contact with others, behavior 4: keep a good distance from other people (at least 1.5 meters), behavior 5: Frequent handwashing with water and soap Adjusted for age, gender, and work history

In their study, Rogers et al. demonstrated that threat-by-efficacy interactions are the fundamental determinants of disease spread ^12^. Witte borrowed two ideas from PMT explained by Rogers et al. The first idea was the structure of a fear appeal which consists of two parts. The first part of a fear appeal identifies a harmful danger existing in the receiver’s environment (severity) and likely to strike (susceptibility). The second part is the efficacy component which identifies such responses as a typical attitude or behavior change. It can help receivers to prevent the threat (response efficacy) ^12^.

Witte showed that receivers are likely to respond to a fear appeal in one of three ways: 1) If the fear and revision message shows a weak threat (low severity and/or susceptibility), no response (attitude or behavior change) will occur. The findings of the present study suggested that 1.38% of health staff were of no-response type. 2) If the message indicates a serious threat (both severe and likely to strike), and the recommended response is effective in threat prevention (high efficacy and response-efficacy), the recipient is expected to be involved in risk control. The results of the current study also pointed out that health staff were at a desirable level of efficiency and threat (86.06%). 3) If the message threat is high, but the recommended response is ineffective (low response effect) and/or out of the list of recipient behaviors (low efficacy), fear control processes will prevail over the danger control process ^14^. In the present study, 2.54% of cases had this situation. The findings denoted that high threat-low efficacy increased health behaviors. This result is consistent with some of the studies conducted based on the EPPM in the fields of self-care behaviors ^15,16^. On the contrary, Roberto et al. (2019) reported that the predicted threat×efficacy interaction was not observed for attitude, intention, or behavior ^14^. Nonetheless, there were main effects for efficacy, but not threat, on just attitude and intentions. The previous studies ^14,17^ have demonstrated that in the EPPM model, the perceived efficacy has a greater impact on the recommended health behaviors, compared to the perceived threat.

The results of the present study showed that based on the reference group (LT/LE), the chance of health behaviors in the LT/HE group was 2-5 times higher, compared to those in the HT/LE group. Therefore, efficacy should be further emphasized for designing and specifying interventions. According to the World Health Organization (WHO) and the Centers for Disease Control and Prevention, the continued use of masks and gloves is essential in all coronavirus care procedures ^18,19^. In this regard, Rajoura et al. reported that 82.6% of physicians and 85% of Indian nurses wore masks at the time of H1N1 influenza pandemic ^20^.

 In the present research, the performance rate of health behaviors recommended by WHO, such as marinating a good distance from other people and frequent hand washing, was not acceptable, even in the HT/HE group. In this regard, only 51.86% and 53.6% of the participants were used to wash their hands regularly and keep a distance of 1.5 meters from others. This could be ascribed to the point that more than 70% of participants (77.3%) were staff of health centers and hospitals. These people had to be in direct contact with patients and service recipients to provide services and patient care. Therefore, it will be very difficult for them to observe a safety distance of 1.5 m. On the other hand, the reason behind the use of gloves and hand sanitizers by 91.5% of participants was their availability in all health care centers. Therefore, this group of participants may have felt less need to wash their hands frequently.

Barnett et al. (2009) showed that in the influenza pandemic, the EPPM provided a useful framework for understanding the basic levels of awareness and willingness to respond to health staff ^17^. It has been found that the continuous use of personal protective equipment by health staff makes them gradually accustomed and eventually satisfied with it^21,22^. To improve the knowledge, attitude, and performance of health staff, it is recommended to perform such measures as continuous supervision, as well as adequate and appropriate training. Among the notable limitations of the present study, we can refer to the use of the self-report method which may have raised the possibility of bias. Moreover, some members of the research community were reluctant to participate in the study.

## Conclusion

 As evidenced by the obtained results, the health staff with low threat and efficacy had a lower rate of the constant exhibition of health behaviors. Therefore, health staff are expected to be at the highest level of threat and efficiency. Moreover, the findings emphasized the effectiveness of the recommended strategies in the prevention of COVID-19 disease. It is hoped that the results of the present study will be of great help to policymakers and public health centers in the development of effective health interventions.

## Acknowledgements

 The authors' deepest appreciation goes to Hamadan University of Medical Sciences. The present study was approved by the Ethical Committee of Hamadan University of Medical Sciences (IR.UMSHA.REC.1398.1092 and 9812209845).

## Conflict of interest

 All authors declare that they have no conflict of interest regarding the publication of the current study.

## Funding

 The current research project did not receive any grant from any organization.

## Highlights

- The results showed that prevention behaviors, such as wearing gloves, using masks, and avoiding contact with others were at a desirable level.
- The health staff with low threat and efficacy had a lower rate of constant exhibition of health behaviors.
- The results of the present study can be of great help to policy makers and public health in the development of effective health interventions.
